# Atypical Antipsychotics in the Treatment of Acute Bipolar Depression with Mixed Features: A Systematic Review and Exploratory Meta-Analysis of Placebo-Controlled Clinical Trials

**DOI:** 10.3390/ijms17020241

**Published:** 2016-02-16

**Authors:** Michele Fornaro, Brendon Stubbs, Domenico De Berardis, Giampaolo Perna, Alessandro Valchera, Nicola Veronese, Marco Solmi, Licínia Ganança

**Affiliations:** 1 New York Psychiatric Institute, Columbia University, New York City, NY 10032, USA; 2 Health Service and Population Research Department, Institute of Psychiatry, King’s College London, De Crespigny Park, London SE5 8AF, UK; brendon.stubbs@kcl.ac.uk; 3 Physiotherapy Department, South London and Maudsley NHS Foundation Trust, London SE5 8AZ, UK; 4 National Health Service, Department of Mental Health, Psychiatric Service of Diagnosis and Treatment, Hospital. Mazzini, ASL 4, Teramo 64100, Italy; dodebera@aliceposta.it; 5 Department of Clinical Neurosciences, Hermanas Hospitalarias—Villa San Benedetto Menni Hospital, FoRiPsi 22032, Italy; pernagp@gmail.com; 6 Hermanas Hospitalarias—Villa San Giuseppe, Ascoli Piceno 63100, Italy; a.valchera@ospedaliere.it; 7 Department of Medicine (DIMED), University of Padua, Padova 35121, Italy; ilmannato@gmail.com; 8 Department of Neurosciences, University of Padua, Padova 35121, Italy; marco.solmi83@gmail.com; 9 Departamento de Psiquiatria e Saúde Mental, Faculdade de Medicina, Universidade de Lisboa, Lisbon 1649-035, Portugal; lg2733@cumc.columbia.edu

**Keywords:** second generation antipsychotic (SGA), mixed features, diagnostic and statistical manual for mental disorders fifth edition (DSM-5), acute bipolar depression, systematic-review, meta-analysis

## Abstract

Evidence supporting the use of second generation antipsychotics (SGAs) in the treatment of acute depression with mixed features (MFs) associated with bipolar disorder (BD) is scarce and equivocal. Therefore, we conducted a systematic review and preliminary meta-analysis investigating SGAs in the treatment of acute BD depression with MFs. Two authors independently searched major electronic databases from 1990 until September 2015 for randomized (placebo-) controlled trials (RCTs) or open-label clinical trials investigating the efficacy of SGAs in the treatment of acute bipolar depression with MFs. A random-effect meta-analysis calculating the standardized mean difference (SMD) between SGA and placebo for the mean baseline to endpoint change in depression as well as manic symptoms score was computed based on 95% confidence intervals (CI). Six RCTs and one open-label placebo-controlled studies (including *post-hoc* reports) representing 1023 patients were included. Participants received either ziprasidone, olanzapine, lurasidone, quetiapine or asenapine for an average of 6.5 weeks across the included studies. Meta-analysis with Duval and Tweedie adjustment for publication bias demonstrated that SGA resulted in significant improvements of (hypo-)manic symptoms of bipolar mixed depression as assessed by the means of the total scores of the Young Mania Rating Scale (YMRS) (SMD −0.74, 95% CI −1.20 to −0.28, *n* SGA = 907, control = 652). Meta-analysis demonstrated that participants in receipt of SGA (*n* = 979) experienced a large improvement in the Montgomery–Åsberg Depression Rating Scale (MADRS) scores (SMD −1.08, 95% CI −1.35 to −0.81, *p* < 0.001) *vs.* placebo (*n* = 678). Publication and measurement biases and relative paucity of studies. Overall, SGAs appear to offer favorable improvements in MADRS and YMRS scores *vs.* placebo. Nevertheless, given the preliminary nature of the present report, additional original studies are required to allow more reliable and clinically definitive conclusions.

## 1. Introduction

Since their introduction, the number of second-generation antipsychotic (SGA) drugs prescribed to bipolar disorder (BD) patients appears to have steadily increased across the worldwide [1,2,3]. Reasons for the observed prescription trend seem to be primarily driven by a corresponding increase in the number of novel SGAs made available, by additional in-label and guidelines indications, and possibly by an in increase in the awareness of BD prevalence and incidence too by the prescribing clinicians [4,5,6,7]. Nonetheless, BD encompasses heterogeneous clinical presentations, including bipolar depression associated with mixed features, which represent a hard to capture diagnostic dimension associated with significant burden, often worsened by improper pharmacological management [8]. The actual prevalence of bipolar depression with mixed features is also remarkable. The Diagnostic and Statistical Manual Fourth Edition (DSM-IV) [9] manic symptoms may occur frequently during and index episode of bipolar depression, with only 31.2% of the cases lacking of any manic symptoms in contrast to up to 54% of the cases presenting with sub-syndromal (14.8%) or full-blown mania [10]. More recently, similar trends have been documented in mood disorder patients based on the Diagnostic and Statistical Manual for Mental Disorder, Fifth Edition (DSM-5) [11], with up to 34% BD Type-I (BD-I) and 33.8% BD Type-II (BD-II) cases experiencing a current Major Depressive Episode (MDE) fulfilling the criteria for the “mixed features” (MFs) specifier *vs.* 26% of Major Depressive Disorder (MDD) cases with associated MFs [12]. Further higher figures of MFs associated to bipolar depression could nonetheless be expected in the clinical setting, including primary care setting [7], considering that the validity of the DSM-5 codes for MFs themselves has been questioned [12,13,14,15,16]. This with special reference towards the DSM-5 exclusion of overlapping symptoms of “unipolar” rather than bipolar depression with MFs, namely “psychomotor agitation”, “distractibility” and “impulsivity”. Indeed, albeit representing trans-nosological features, such excluded features of bipolar and unipolar depression are nonetheless perceived as the actual differential diagnostic features by many clinicians [17,18,19,20,21,22]. Taken altogether, these issues point to the compelling need for both an enhanced recognition and discriminant validity of bipolar MFs [23,24,25,26] as well as a more effective, evidence-based pharmacological treatment, with a special emphasis towards SGAs [27,28].

To the best of our knowledge, the present meta-analysis of (placebo)-controlled clinical trials (RCTs) represents the first preliminary report investigating the use of SGAs in the treatment of MFs in BD.

## 2. Results

### 2.1. Included Studies

**Figure 1:**
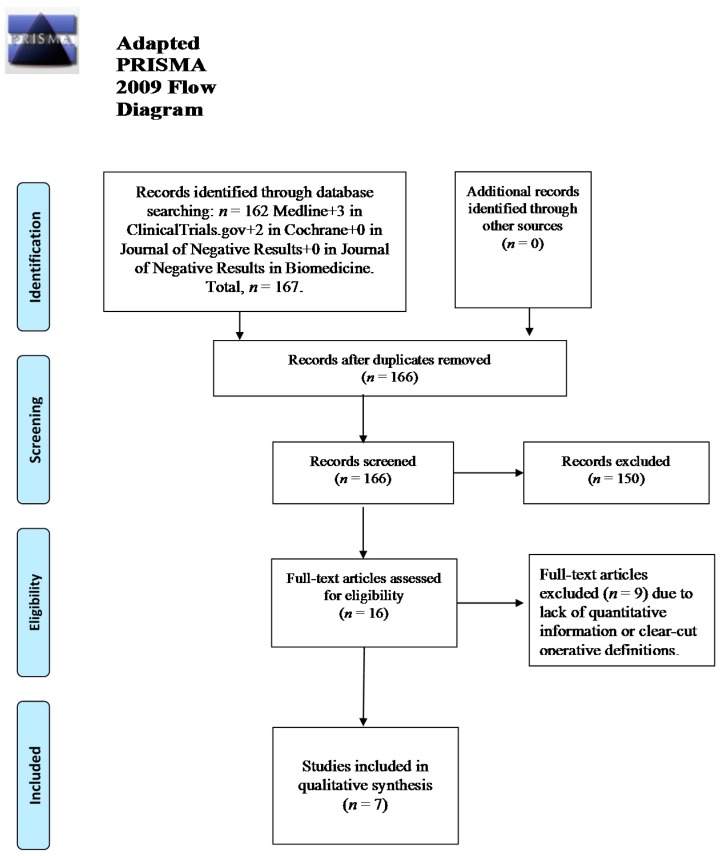
Flowchart of the study.

Details about the multi-step screening of results have been outlined in Figure 1. Only eight articles, including seven unique studies (including *post-hoc* of overlapping samples) were identified that met our inclusion criteria. Six out of the seven included results were double-blind, placebo-controlled trials [14,29,30,31,32,33,34]. Overall, the methodological quality of the randomized controlled studies was good, scoring ≥3 according to the Jadad scale [35]. Only one trial was a placebo-control study of an SGA augmentation, actually olanzapine with the antidepressant fluoxetine [32], which had a Newcastle–Ottawa (NCO) scale total score of eight (good quality) [36]. Owing to the presence of *post-hoc* studies based on the same sample(s): [33] and [34]; [32] and [14], the cumulative results, and the essential demographic and clinical information as outlined in Table 1 reflects six original results.

**Table 1:** Essential characteristics of the studies included in the analysis.

Study	SGA	Mean Baseline/Study Endpoint Dose (Range) in mg	Duration of the Study	SGA Group				Placebo Group			
				MADRS Baseline	MADRS Endpoint	YMRS Baseline	YMRS Endpoint	MADRS Baseline	MADRS Endpoint	YMRS Baseline	YMRS Endpoint
* Patkar A. *et al.;* 2012 [33]	Ziprasidone	40/129.7 (80–160)	6-week	23.4 ± 6.5	12.0 ± 10.9	8.4 ± 6.1	4.7 ± 5.2	25.1 ± 7.9	19.12 ± 9.3	8.8 ± 6.2	6.5 ± 5.1
&											
* Pae C.U. *et al.*, 2012 [34]											
Sherwood B. *et al.*, 2014 [30]	Quetiapine	50/600 (50–600)	12-week	18.6 ± 7.0	Undisclosed (not an outcome measure)	13.9 ± 6.7	Undisclosed (not an outcome measure)	25 ± 9.2	Undisclosed (not an outcome measure)	13.6 ± 8.2	Undisclosed (not an outcome measure)
* Tohen M. *et al.*, 2014 [14]	Olanzapine	5/20 (5–20)	6-week	31.4 ± 6.0	26.14 ± 4.0	4.61 ± 2.8	2.55 ± 1.8	30.53 ± 6.2	28.5 ± 4.2	4.94 ± 1.36	4.62 ± 2.5
&											
* Benazzi F. *et al.*, 2009 [32]											
McIntyre R.S. *et al.*, 2015 [29]	Lurasidone	20/20–60 (mean endpoint dose = 31.8) (20–60)	6-week	31.2 ± 5.2	15.5 ± 3.4	5.9 ± 2.2	3.5 ± 2.9	31.2 ± 5.3	20.3 ± 2.1	6.2 ± 2.6	3.9 ± 1.3
		&									
		20/80–120 (mean endpoint dose = 82) (80–120)									
Berk M. *et al.*, 2015 [31]	Asenapine arm	20/10-20 (10–20)	3-week	24.64 ± 3.73	13.89 ± 3.7	27.52 ± 4.79	10.94 ± 2.4	26.23 ± 4.86	21.04 ± 3.1	27.19 ± 4.79	16.92 ± 2.6
	Olanzapine arm	15 (5–20)	3-week	25.03 ± 4.33	16.58 ± 3.32	28.36 ± 6.49	Undisclosed				

* *Post-hoc* reports. SGA = Second Generation Antipsychotic; MADRS = Montgomery–Åsberg Depression Rating Scale [37]; YMRS = Young Mania Rating Scale [38].

### 2.2. Efficacy

Based on our inclusion criteria, the efficacy of the SGA in mixed depression varied across different studies. Ziprasidone proved to be significantly superior *vs.* placebo (*p* = 0.0038) in a 73-patient, 6-week, 2012 randomized trial [33]. However, a 72-patient, 6-week, 2011 randomized report [34] did not find an effect, despite an equivalent mean daily dose = 129.7 ± 45.3 mg of ziprasidone *vs.* placebo (126.1 ± 47.1 mg) in both studies. Similarly, asenapine was associated to a significantly greater reduction in depressive symptoms assessed by the means of the MADRS when compared to placebo (*p* = 0.0195) and olanzapine (*p* = 0.0436) at all study points [31]. Olanzapine monotherapy was nonetheless superior over placebo based on pooled data from 2 placebo-controlled trials (least-squares mean differences between olanzapine and placebo in the change of MADRS total scores were −3.76 (*p* = 0.002), −3.20 (*p* = 0.001), and −3.44 (*p* = 0.002) for mixed features *n* = 0, 1, 2, or 3 respectively) [14]. Response rates of olanzapine/fluoxetine combination in mixed features acute BD-I depression *vs.* olanzapine monotherapy *vs.* placebo were OR (odd ratio) = 2.00 (95% CI, 0.96–4.19) and OR = 3.91 (95% CI, 1.80–8.49) respectively [32].

Lurasidone *vs.* placebo was also associated to significant reduction of the MADRS scores both in acute bipolar patients with (−15.7 *vs.* −10.9; *p* = 0.001; mixed model for repeated measure effect size = 0.48) or without mixed features (−15.2 *vs.* −10.8; *p* = 0.002; mixed model for repeated measure effect size = 0.48) over a six-week follow-up [29]. Aripiprazole as monotherapy or augmentation treatment was also documented to be superior over placebo in terms of efficacy, though no clear-cut quantitative measure was provided about this outcome [39]. On the contrary, quetiapine did not reduce alcohol consumption in patients with either BD and/or depressive mixed phase and alcohol consumption when compared against placebo [30].

### 2.3. Meta-Analysis

#### 2.3.1. Young Mania Rating Scale (YMRS)

**Figure 2:**
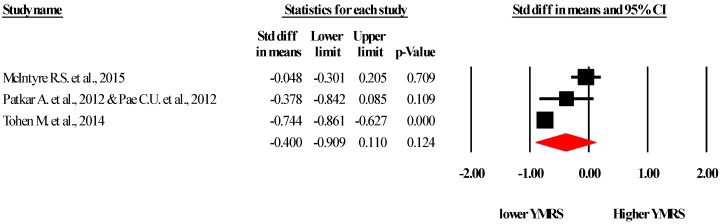
Forest plot of the Young Mania Rating Scale (YMRS) across the included studies. CI = confidence intervals. Referenced studies: McIntyre R.S. *et al*., 2015 [29]; Patkar A. *et al.*, 2012 [33] & Pae C.U. *et al.*, 2012 [34].

It was possible to pool data from three studies [14,29,33] investigating the YMRS [38] including 907 allocated to SGA and 652 allocated in to the control arm. The pooled analysis demonstrated that SGA decreased YMRS scores (SMD (Standardized Mean Difference) −0.40, 95% CI −0.90 to 0.11, I2 = 91) although this was not significant (*p* = 0.12) see Figure 2. The Duval and Tweedie trim and fill analysis adjusting for publication bias, trimmed two studies to correct for one outlier, offering a new effect size (SMD −0.74, 95% CI −1.20 to −0.28) which then became significant.

#### 2.3.2. Montgomery–Åsberg Depression Rating Scale (MADRS)

**Figure 3:**
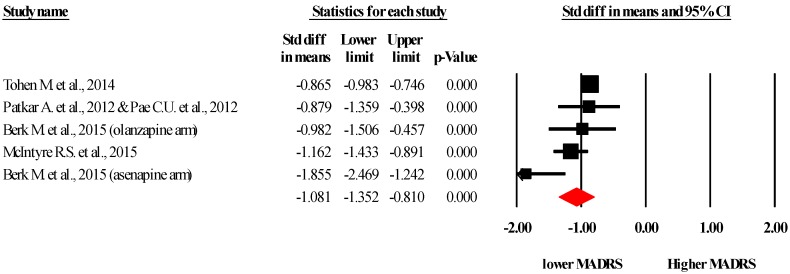
Forest plot of the Montgomery–Åsberg Depression Rating Scale (MADRS) across the included studies. Referenced studies: Tohen M. *et al.*, 2014 [14]; Patkar A. *et al.*, 2012 [33] & Pae C.U. *et al.*, 2012 [34]; Berk M. *et al.*, 2015 [31]; McIntyre R.S. *et al*., 2015 [29].

Five study *arms* across *four* unique studies [14,29,31,33] provided data for the influence of SGA on MADRS scores. Pooled data from 979 people taking SGA and 678 controls established that SGA resulted in a large and significant reduction in MADRS scores *vs.* placebo (SMD −1.08, 95% CI −1.35 to −0.81, *p* < 0.001, I2 = 68%) see Figure 3. Upon calculation of the Duval and Tweedie trim and fill meta-analysis, one study was trimmed and the effect size marginally increased (SMD −1.17, 95% CI −1.52 to −0.85) confirming the beneficial effect of SGA on MADRS scores.

There was insufficient data to meta-analyze other outcome measures.

## 3. Discussion

Our study found that there is some early and promising evidence for the use of SGA to improve BD depression in people with MFs. However, the relatively small number of studies and other caveats clearly preclude a more definitive conclusion at this stage.

### 3.1. Main Results and Implication for the Clinical Practice

The results from our exploratory meta-analysis results are encouraging, demonstrating that SGA results in significant and large improvements in MADRS scores (SMD 1.08, 95% CI −1.35 to −0.81, *p* < 0.001). Moreover, following the adjustment of potential publication bias, our pooled analysis including 1552 participants demonstrated that SGA result in significant improvements in YMRS scores (SMD −0.74, 95% CI −1.20 to −0.28).

Clearly, further RCT studies are warranted in order to build upon and shed further light on the efficacy and role of different SGAs in the treatment of acute bipolar depression with MFs. Nonetheless, we submit that greater attention should be paid over the actual psychopathological predictive value of the MFs themselves, ideally aiding the prescribing clinician towards a more patient-tailored pharmacological management [40], enhanced treatment adherence [41,42], reduced need for polypharmacy in BD [43,44] and reduced chances of treatment resistance overall [45]. In addition, systematic standardized interviews to assist with the diagnostic codes and course specifiers of bipolar depression are needed, as it is likely that patients with ultra-ultra-rapid cycling may be incorrectly identified as having mixed features when in reality they cycle from one polarity to another within the course of a day and represent a different illness phenotype [46].

Specifically, the actual validity of the MFs constructs according to the DSM-5 has been questioned, as afore mentioned in the present text. This is a key reason why the present report did not solely rely on the narrow DSM-5 criteria for MFs. Therefore, it is of primary relevance to rank different MFs against each other, especially with regard to the “*absence of increased activity*” as a potential effect modification predicting favorable response at study endpoint, despite the trans-nosological distribution of those “overlapping” BD/MDD “polythetic” diagnostic criteria excluded by the DSM-5 as MFs (namely, “psychomotor agitation”, “distractibility” and “impulsivity”) [47].

Indeed, “absence of increased activity” does not necessarily mean “presence of decreased” levels of activity, since those cases without increased activity may also encompass people with “normal” levels of activity too. The implication for differential diagnosis of bipolar *vs.* “unipolar” depression with MFs are of primary relevance to the clinical practice, as briefly further discussed over in the text.

As recently prompted out [15,40,48], the claimed inaccuracy of the DSM-5 criteria for MFs should represent a crucial issue in the approach of future large-sampled RCTs ideally assessing the efficacy and cost-effectiveness of the accounted SGAs, ideally focusing on long-acting agents too [41], in the treatment of BD as well as unipolar [49] depression with MFs, beyond the boundary of the sole DSM-5 criteria.

### 3.2. Limitations of the Study

The results presented here should be interpreted as preliminary, and there are a number of limitations, which are reflected by those within the primary data. Among others, both publication and “apples and oranges” (heterogeneous SGAs) biases must be accounted in the interpretation of the preliminary results coming from the present meta-analysis. Similarly, most of the included studies were *post-hoc* reports since the original studies were conducted before the proposed (or final) diagnostic changes of the DSM-5 were made available. Among other implications, the samples included from the original studies did not represent complete randomized populations. Also data obtained from open-label and double-blind studies were combined, and it was impossible to discern between unipolar and bipolar cases of depression with MFs in one the (open-label) studies included in our analyses [33]. The SGAs have heterogeneous pharmacodynamics and pharmacokinetics profiles. The same SGA could also provide different clinical effects depending on the dose on the clinical parameters (e.g., cognitive status, alcohol use comorbidity) across varying studies.

In addiction, only three out six of the original studies included in the present analyses provided clear-cut data about the YMRS total scores at study endpoint. While the overall quantitative information available on the matter was nonetheless sufficient to allow a preliminary extraction, this may nonetheless have hampered the actual validity of overall results. Moreover, the present study did not rely just on the operational definition of MFs as coded by the DSM-5, as it was due also to the broader and/or alternative operational definitions as documented across the included studies. While this latter issue could be perceived as a “measurement bias”, we nonetheless submit that this would allow a better perception of “real-world” routine clinical practice diagnostic choices. This is also why some of the original studies included in the present meta-analysis accounted for “psychomotor agitation” (or possibly “impulsivity” and “distractibility”), despite that such features were not officially accounted as depressive MFs by the DSM-5. In fact, while these latter “overlapping” trans-nosological features were excluded by the DSM-5 [15], their likely discriminant validity has nonetheless been stressed by many clinicians [25,48,50], even with regard to SGA-treatment response in acute bipolar depression [51], thus supporting the need for a broader operational definition of MFs beyond the sole DSM-5 specifiers.

Similarly, though heterogeneous in terms of pharmacodynamics, pharmacokinetic and dose-effect profiles, we submit that the accounted SGAs share a common clinical and evidence-based “backbone”, which could allow a pooled extraction. Indeed, as little as two studies should be acceptable for a preliminary meta-analytic report, even when accounting for heterogeneous active compounds in the same class of drugs and/or differential doses [52], whereas those meta-analysis accounting for substantially heterogeneous compounds and/or based on incomplete sources would not be informative not even if including a high number of studies [53].

In conclusion, the preliminary data suggests that SGA may have a potential role in improving symptoms among people with MFs and BD depression.

## 4. Materials and Methods

### 4.1. Data Source and Methods of Search

The present meta-analysis adhered to the Meta-analysis Of Observational Studies in Epidemiology (MOOSE) guidelines [54].

A systematic search was conducted using MEDLINE database (www.medline.com) from January 1990 to September 2015 for randomized or open-label placebo-controlled trials of SGA(s) in the treatment of MFs associated with acute bipolar depression in adults. Searches were also conducted using ClinicalTrials.gov (https://clinicaltrials.gov/), Cochrane library (http://www.cochranelibrary.com/) and the Journal of Negative Results (www.jnr-eeb.org), the Journal of Negative Results in Biomedicine (www.jnrbm.com). Adjunctive therapies of SGA with antidepressant(s) or mood-stabilizers were also considered. Articles considering MFs defined according to the DSM-5 criteria, or operatively extracted based on selected items of the major rating scales as reported in the methodological sections documented across the studies using the DSM-IV diagnostic codes were included. Only English language studies were considered. 

Owing to the relatively recent introduction of SGA compounds compared to other established psychotropic medication, two independent authors appointed for data identification and selection (Michele Fornaro and Licínia Ganança) limited the covered period from year 1990 to writing time (September 2015). The search terms used were “second generation antipsychotics”, “atypical antipsychotics”, “acute bipolar depression”, “mixed features”, “mixed episodes”, “depression”, “dysphoric depression”, “agitated depression”, or their combination. The reference lists of all included articles were also considered. When required, we attempted to contact the authors to gather additional information about specific MFs, if this was not clear in the paper or if we required additional data.

Both auto- and hand-searches for “type-I” (“duplicates among/across different databases”) and “type-II” (duplicate publications in different Journals/issues) [55] were performed using Thomson Reuters Endnote X7™ for Microsoft Windows™ [56]. Finally, the included studies were further assessed for quality according to the Jadad scale [35].

### 4.2. Data Analysis

A random effects model was set “a priori” to perform our analyses. This approach was essentially due to the heterogeneous characteristics of the studies at inclusion, namely “duration of the trial”, SGA compound (and dose), clinical sample composition and patients’ characteristics, as well as the primary outcome measures adopted across varying sources. By accounting for both within- and between-study variance, a random-effect model allows one to estimate the average effects of treatment(s), thus representing a preferred methodological approach to analyze studies characterized by remarkable heterogeneity of the adopted methods. We used the following primary efficacy measures: changes in the total scores of the Montgomery–Åsberg Depression Rating Scale (MADRS) [37] and/or the Hamilton Scale for Depression 17-item version (HAM-D17) [57] for depressive symptoms and the Young Mania Rating Scale (YMRS/MRS) [38] for (hypo-)manic symptoms. Approximation of the HAM-D17 scores to the corresponding MADRS approximated values was planned when needed using conventional guidance [58]. Manic features of (bipolar) depression with MFs were operatively defined using the method adopted by Tohen M. *et al.* (2014), based on the number of the concurrent manic symptoms recorded at baseline (0, 1 or 2, ≥3), and measured by the YMRS items referring to the DSM-5 criteria. Specifically, the following YMRS items were regarded: item 1 (“elevated, expansive mood”); item 2 (“increased motor activity/energy”); item 4 (“decreased need for sleep”); item 6 (“pressure to speech”); item 7 (“flight of ideas; racing thoughts”) and 11 (“insight”).

An alternative definition of MFs within the course of bipolar depression was also considered in accordance with Patkar A. *et al.* (2012), based on the presence of “two or three” DSM-IV-defined manic symptoms during the course of a DSM-IV-defined MDE [33]. We used mean change in YMRS of MRS from baseline to endpoint defined in each of the included studies as primary outcome measure. The standard mean differences (SMD) was calculated with 95% confidence interval (CI). All analyses were conducted using Comprehensive Meta-analysis version 3 software [59]. For those studies presenting results in the mean standard error (SE) form, the SE was converted to standard deviation (SD) using the formula: SE=SD/√n, where *n* is the sample size. Heterogeneity was assessed by the I2 statistic [60]. For each pooled analysis, we conducted a trim and fill adjusted analysis to adjust for publication bias [61]. The trim and fill analysis provides an estimation of the number of studies missing from a meta-analysis due to the deletion of the most extreme results on one side of the funnel plot. A new effect size is recalculated until the funnel plot is symmetric.

### 4.3. Essential Description of the Main Rating Scales and Their Scoring:

HAM-D [57]: The scale is cornerstone tool for the assessment of depression. The HAM-D includes 21 items, yet the scoring is based on the first 17. A score of 0–7 is considered to be “normal”. Scores of 20 or higher indicate “moderate” or more “severe” forms depression, and are usually required for entry into clinical trials.

Jadad scale [35]: it is a quick procedure originally developed by Dr. Alejandro Jadad-Bechara (2007) to assess the quality of (randomized) clinical trial. Scores equal or greater than 3 indicate good quality.

MADRS [37]: Is a 10-item questionnaire aimed at measuring the severity of depressive episodes in patients with mood disorders, including bipolar patients with current mood depression. Compared to other scales developed for the assessment of the severity of depression, the MADRS is usually preferred in case of pharmacological clinical trials, as it is sensitive towards changes in depression severity eventually induced by active drug(s) or placebo. The questionnaire includes questions on the following symptoms 1. “Apparent sadness” 2. “Reported sadness” 3. “Inner tension” 4. “Reduced sleep” 5. “Reduced appetite” 6. “Concentration difficulties” 7. “Lassitude” 8. “Inability to feel” 9. “Pessimistic thoughts” 10. “Suicidal thoughts.” Usual cutoff points are: 0 to 6—“normal /symptom absent”; 7 to 19—“mild depression”; 20 to 34—“moderate depression”; ≥34—“severe depression”.

NCO [36]: The Newcastle–Ottawa Scale is used for the assessment of the quality of non-randomized studies in meta-analyses. Higher scores indicate better quality (e.g., a score of 8 indicates good quality).

YMRS/MRS [38]: The scale comprises 11 questions used to measure the severity of manic episodes. The scores from each question are added together to form a total score ranging from 0 to 60, with higher scores indicating a greater severity of symptoms. A score of 12 or higher indicates a potential case of mania or hypomania, while a score of 21 or above indicates a probable case. Most “depressed” patients have a total score of about 3 at the YMRS, while most euthymic cases score about 2. Yet, the typical YMRS baseline scores can vary a lot in clinical trials. Sometimes a clinical study entry requirement of YMRS >20 generates a mean YMRS baseline of about 30.

## Abbreviations

BD: Bipolar Disorder; either Type-I (BD-I) or Type-II (BD-II)
CI: Confidence Interval
DSM: Diagnostic and Statistical Manual for Mental Disorder
DSM-IV: DSM, Fourth Edition
DSM-5: DSM, Fifth Edition
HAM-D: Hamilton Scale for Depression [57]; HAM-D-17 = HAM-D, 17-item version [57]
Jadad (scale): a rating scale developed by Dr. Jadad (2007)
MADRS: Montgomery–Åsberg Depression Rating Scale [37]
MDD: Major Depressive Disorder
MDE: Major Depressive Episode
MEDLINE: Medical Literature Analysis and Retrieval System Online (U.S. National Library of Medicine’s life science database)
MFs: Mixed Feature(s) (of the DSM-5)
MOOSE: Meta-analysis Of Observational Studies in Epidemiology [guidelines]
MRS: Mania Rating Scale (see also “YMRS”—Authored by Young R.C. *et al.*, 1978) [38]
NCO: Newcastle Ottawa Scale
NOS: Not Otherwise Specified (referred to the DSM-IV. Note: the DSM-5 essentially replaced “NOS” with two categories, either “other specified disorder” or “unspecified disorder”; a new category, namely “NEC”—“not elsewhere classified” was also introduced by the DSM-5 [62])
O.R.: Odd ratio
RCT: Randomized Controlled Trial
S.D.: Standard Deviation
SGA: Second Generation Antipsychotic
SMD: Standardized Mean Difference
YMRS: Young Mania Rating Scale [38]
